# A-Cross: a predictive model for estimating allergen cross-contact in dry food production

**DOI:** 10.1186/2045-7022-5-S3-P143

**Published:** 2015-03-30

**Authors:** Ben Remington, Juliën Voogt, Rene Crevel, Daryl Cromie, Astrid Kruizinga, Niels Lucas Luijckx

**Affiliations:** 1 TNO, Zeist, The Netherlands; 2 Unilever, Milton Keynes, United Kingdom

## 

Food products may contain unintended allergens due to cross-contact through the production process. This can pose a risk to allergic consumers. Food producers are aware of this and optimize production to control the likelihood and extent of cross-contact as much as possible. To ensure the safety of allergic consumers and determine the need for mitigating measures, the residual risk must be assessed. A model to track and predict the concentration of unintended food allergens in a product during the production of dry food powders was developed, based on modelling individual components of the production process. The model is referred to as the A-Cross model and is currently in prototype status.

In an effort to protect allergic consumers, food producers will optimize their production processes to control the risk of cross-contact as much as possible. Previous tools have been developed for allergen risk assessment and risk management. However all involve costly ingredient and final product analysis, that by definition are momentary glimpses into production.

The predictive A-Cross model was developed using milk as the prototype allergen. TNO expertise was combined with practical information from Unilever, available scientific literature and controlled sample analyses to develop the prototype. This enables companies to assess and address the likelihood of allergen contamination from individual process units (weighing, transport, mixing, etc.). It can also be used to rapidly examine different scenarios (for instance, different scheduling sequences) and establish the optimum ones with minimal or no testing.

In a second development phase of the A-Cross model, TNO, Unilever and Intertaste (The Netherlands) aim to extend and verify the A-Cross model across other production processes, other allergens, and other ingredients. A scientifically sound and verified model for allergen cross contamination in food production acilities will benefit all food allergy stakeholders.

